# Effect of WhatsApp-based BETTER model sexual counselling on sexual function and sexual quality of life in breast cancer survivors: a randomized control trial

**DOI:** 10.1186/s12905-024-03283-w

**Published:** 2024-08-09

**Authors:** Shirin Nazarzadeh, Fatemeh Moghaddam-Tabrizi, Mahmonir Haghighi, Rasool Gharaaghaji-Asl

**Affiliations:** 1 Department of Consultation on Midwifery, School of Nursing and Midwifery, Urmia University of Medical Sciences, Urmia, Iran; 2 Reproductive Health Research Center, Clinical Research Institute, Urmia University of Medical Sciemces, Urmia, Iran; 3 Department of Psychiatry, School of Medicine, Razi Hospital, Urmia University of Medical Sciences, Urmia, Iran; 4 Department of Epidemiology and Biostatistics, School of Medicine, Urmia University of Medical Sciences, Urmia, Iran; 5 UMSU Central Site. Orjhans Street, Resalat BLvd., Urmia, Postal Code: 571478334 Iran

**Keywords:** Sex counselling, Sexual function, Sexual quality of life, Breast cancer, Randomized control trial

## Abstract

**Objective:**

The aim of the study was to determine the effect of WhatsApp-based BETTER sex counselling on sexual function and sexual quality of life in breast cancer survivors in a randomized control trial.

**Methods:**

This is a randomized controlled trial in which a total of 90 breast cancer survivors were recruited using convenience sampling and then randomly assigned to two groups of WhatsApp-based BETTER model counselling and routine care. Data collection tools consisted of a demographic questionnaire, the Sexual Quality of Life-Female (SQOL-F) and the Sexual Function Index (FSFI-BC). Participants in the intervention group were given access to the 6-week program. The program consisted of six consultation and assignment packages covering all six steps of the BETTER model. Data were analyzed using SPSS software version 20. Chi-square test, independent samples t-test and repeated measures analysis of variance were used. The significance level (*p*-value) was considered to be less than 0.05.

**Results:**

In the control group, the mean score of SQL scale changed from 35.16 ± 10.71 to 35.16 ± 12.97 (*P* > 0.05) and in the intervention group, it significantly increased from 34.76 ± 10.13 to 68.20 ± 20.48 (*P* < 0.001). Similarly, the comparison of mean of FSF in the control group showed a none-significant change from 58.13 ± 7.11 to 58.35 ± 6.11 (*P* > 0.05), and in the intervention group, it significantly improved from 59.49 ± 6.10 to 120.73 ± 25.54 (*P* < 0.001). The results of rANOVA indicated that there was a significant difference in the mean scores of the SQL and SFS between the two groups from pre- to post-intervention, and then over the 1-month follow-up period in the intervention group (*p* < 0.001). Considering partial eta squared, the effect of the intervention had the highest interaction effect on both variables of the sexual function index (η2 = 0.73) and sexual quality of life (η2 = 0.41).

**Conclusions:**

The intervention program was a successful model for improving female sexual quality of life and female sexual function in breast cancer survivors.

**Trial registration:**

IRCT20210926052601N1, 7–11-2021.

## Background

Breast cancer is the most common form of cancer and the second leading cause of death in women around the world [1]. Breast cancer kills 42,260 people annually, 41,760 of whom are women. One in eight US women will develop invasive breast cancer in her life [2]. Iranian women with breast cancer are relatively younger than their Western counterparts, although the incidence of breast cancer has increased worldwide over the past four decades. Breast cancer age in Iran is at least 10 years lower than developed countries [3]. The average age of Iranian women at diagnosis is 46–49 years [4], while 74 percent of the average age at cancer diagnosis were more than 50 years in England [5].

Breast cancer causes psychological and social problems by causing changes in this organ, which is a symbol of a woman's femininity and sexuality. Studies show that affected women often express dissatisfaction after a mastectomy [6, 7]. Following primary treatment for breast cancer, women face not only the stigma associated with cancer, but also changes in sexuality, femininity and fertility [8]. Sexual dysfunction is the most common long-term breast cancer-related problem affecting women's sexual quality of life and can occur at any time from diagnosis, during treatment, and for years after treatment. Multiple linear regression analysis showed that the sexual quality of life of breast cancer survivors was significantly correlated with their sexual function [9].

Neglecting these sexual problems in patients causes distress and further problems in relationships. It affects all aspects of a person's life [10]. The use of sexual counseling methods helps patients maintain their emotional relationship with their spouse and have a good sex life, despite all the physical and mental changes associated with their disease [11]. The BETTER model is one of the sexual counselling models and includes 'Bring up', 'Explain', 'Share resources', 'Time', 'Educate' and 'Record' parts and was first introduced by Cohen et al. in 2004 and it is also reported that interviews based on the BETTER model reduce stress and anxiety in sexual relationships [12, 13].

From an Asian perspective, a study by Maleki et al. in Iran explained how women's ability to discuss sexual issues is limited by their passive role in sexual relationships. In Muslim communities, there is a belief that women are responsible for satisfying their male partners and are less concerned with their own sexual satisfaction. Their study perfectly captures the complexity of sexual life and the cultural barriers involved. It explains how, in addition to the already existing physical complications of sexual function and breast cancer, women in societies like Iran are usually under considerable pressure, feel incompetent, and suffer from emotional problems [14]. Other studies also showed the sexual problems cause feelings of shame and guilt, and such issues are taboo in the culture of the present study [14, 15], so couples often have difficulty expressing sexual problems to others and are consequently deprived of face-to-face counselling services. On the other hand, ignorance about sexual and reproductive health is contrary to women's empowerment and violates these human rights [16].

Online has advantages over face-to-face contact. One of the benefits of WhatsApp-based education is to increase access to education, improve self-efficacy, knowledge production, cost-effectiveness, learner flexibility and interaction [17]. For some people, the anonymity that online counselling sometimes provides may make it a preferable option to face-to-face therapy. Therefore, the present study aimed to investigate the effect of WhatsApp-based sexual counselling using the BETTER model on sexual quality of life and sexual function in breast cancer survivors.

## Methods

### Aim

The aim of the study is to determine the effect of WhatsApp-based counselling using the BETTER sex model on female sexual quality of life and female sexual function in breast cancer survivors in 2022.

### Study design

Randomized controlled trial with consenting participants allocated to intervention or routine self-care. Participants completed questionnaires prior to, immediately after and one month after the intervention. All questionnaires were self-administered.

### Participants

Eligible participants were those diagnosed with stage I, II, IIIa breast cancer (according to the diagnostic result in the participants' documents) with experience of mastectomy, a minimum of 6 months and a maximum of 5 years since the completion of radiation and chemotherapy, married women living with their spouse, women of reproductive age (18–49 years), not pregnant or breastfeeding, not using drugs or alcohol, not receiving any other education or counselling for their sexual dysfunction, no sexual dysfunction in the spouse according to the individual's statement, and having a smartphone with internet access, being literate and having the husband's consent to participate in the counselling program. Exclusion criteria were the occurrence of any physical or mental illness or accident and psychological trauma affecting sexual performance, pregnancy during the conduct of the study, dropping out of the group, attending other counselling sessions outside the study program.

### Assessments

Information was collected using questionnaires on personal characteristics and disease status, Sexual Quality of Life-Female (SQOL-F) and Female Sexual Function Index-Breast Cancer (FSFI-BC).

### Demographic questionnaire

The demographic questions were participants' age, age at menarche, occupation, level of education, adequacy of family income, number of children, place of residence, and information about participants' disease (duration of cancer detection and stage of disease) and other characteristics listed in Table 2.

### The sexual quality of life-female (SQOL-F) questionnaire

The SQOL-F consists of 18 questions on a six-point Likert scale. The minimum score is 18 and the maximum is 108. A higher score on this questionnaire indicates a higher sexual quality of life. A score of 18 to 36 is considered a low sexual quality of life, a score of 37 to 72 is considered a moderate sexual quality of life, and a score of 73 to 108 is considered a high sexual quality of life [18]. The Persian version of the SQOL-F was validated by Masoumi et al. with a Cronbach's alpha of 0.73 [19].

### Female Sexual Function Index, the *FSFI*-*BC* (FSFI-BC)

The FSFI-BC is a 34-item self-report scale (5- or 6-point Likert scale) with 8 subscales: changes after cancer, libido, arousal, lubrication, orgasm, pain, satisfaction and distress, and additional questions about sexual partners. Four additional items relating to the role of the spouse in sexual dysfunction have been added to aid clinical interpretation. Thirteen items are common to all and the other fifteen items separately assess arousal, orgasm, lubrication and pain in sexually active women. Higher scores on each subscale indicate better sexual functioning. Scores below 15, 18, 12, 12, 9, 9 respectively in the subscales of change after cancer, distress, libido, lubrication, orgasm and pain are interpreted as sexual dysfunction and require follow-up. The two satisfaction items and the additional questions about the sexual partner are only for interpretation and assessment in the clinic and were not scored or assessed by the questionnaire designer in the sexual function study. Five questions about changes after cancer, two questions about libido, and six questions about distress are common to both groups of women with and without sexual activity. The 6-point Likert scale is related to the lubrication subscale in sexually active women [20]. The Persian version of the SQOL-F was validated by Masjoudi et al. with a Cronbach's alpha of 0.81 [21].

### Procedures and randomization

**Fig 1.**
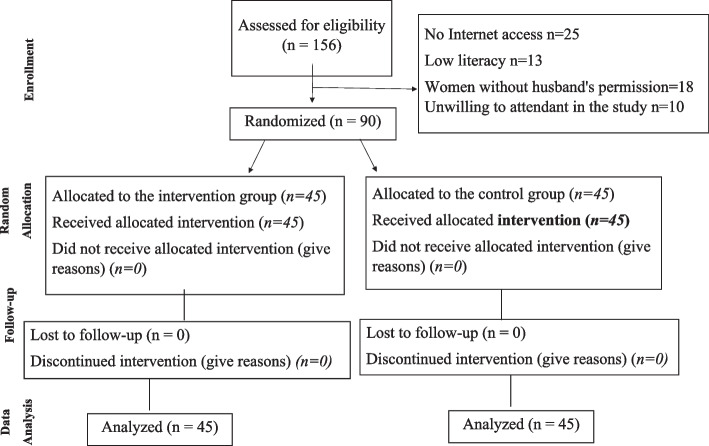
Flow chart of patients inclusion, follow-up, and data analysis

A single-blind, parallel, randomized controlled trial was conducted in 90 women in Iran from November 2021 to March 2022. Sampling was initiated after obtaining the ethical approval code from the Ethics Committee of Urmia University of Medical Sciences (#IR.UMSU.REC.1400.248) and registering the study information with the Registry Center of Clinical Trials (#IRCT20210926052601N1). To conduct the study, the researcher first visited the oncology training and medical centers and visited the breast cancer survivors using convenience sampling. In this regard, 156 survivors were screened for eligibility, so 90 women were eligible & consented. In fact, sixty-six women did not meet the inclusion criteria and were therefore not included in the study (Fig. 1), and participants were then equally allocated to the intervention (Whatsapp-based BETER sex model counselling) and control (routine self-care advice) groups using concealed randomized allocation. This was done by writing the letters A (intervention) and B (control) on 90 cards. Each patient then randomly selected one of the cards and was allocated to the intervention or control group based on the letter written on the card. This part was done by a person who was not involved in the trial (to ensure blinding). The consenting women in each group were then asked to sign the consent form. The pre-test questionnaires including demographic information, FSFI-BC and SQOL-F were then completed by the women and the score obtained was used as the baseline score for comparison. Finally, in the intervention and control groups, all allocated participants were divided into four groups (12–11 women in each WhatsApp group), and to respect the privacy and security of the participants who did not want to reveal their identity (25 participants), 25 SIM carts were given by the researchers.

### Intervention

The intervention program, following all 6 steps of the BETTER Sex model, consisted of 6-week packages of counselling and assignments. (Table 1) [22, 23], through the WhatsApp application as a shared social network in the cultural context of the research community.

**Table 1:** The Whatsapp, Intervention Program Based on the BETTER model delivered in six consecutive steps [24, 25]

Stage One: Bring up During this phase, the trained researcher raised the issue of sexuality. Although she believed that some women would be uncomfortable discussing the subject, by raising the issue she ensured that the women knew that the researcher was willing to discuss it if they were reluctant to express their concerns
Stage Two: Explain The researcher, through an open discussion with the women, explained to them that sexuality is an important and meaningful aspect of their lives. She tried to convey to them that sexual problems can have an impact on women's psychological status and marital satisfaction and expressing the sexual complications and seeking the strategies to overcome the related concerns are their rights, so they should feel less embarrassed and be brave to explore the unspoken words, in this way she struggled to normalize these issues which was considered as a taboo in their culture. The importance of sexual activity and its impact on quality of life was explained in this context
Stage Three: Tell At this stage, the researcher informed the women that if the intervention was not effective in resolving their problems, then they would be referred to another professional who could help to resolve the problem. Some books as appropriate and available sources of information are introduced at this stage Stage Four: Time
The researcher, while respecting the clients' privacy and emphasizing confidentiality, gave them the opportunity to talk about their sexual problems and concerns. In this regard, she asked the clients to use a private line
Stage Five: Educate • At this stage, the researcher delivered appropriate educational packages in the form of recorded voices, pamphlets, pictures, motivational clip arts, films, animations, gifted handbooks by personalized mail to the participants based on the following topics - Female reproductive system and the components of the sexual response cycle - Breast cancer treatment, its possible side effects on the performance of sexual activity - Motivation to practice Kegel exercises, mindfulness techniques, sensation focus exercises to improve intimacy, different technical positions during intercourse and the use of lubricants - Moving towards a better body image by focusing on the positive aspects of the body - Practice eliminating anxiety and stress through the use of relaxation - Techniques such as breathing exercises, taking part in painting and music classes Encouraging regular exercise/walking for at least 30 min and going to the gym Encouraging a diet rich in fruit and vegetables - Recommendations for reducing symptoms of breast cancer treatment side effects such as nausea, vomiting, diarrhea, dyspnea, gingivitis with herbal remedies - Introduce some energy conservation strategies to help overcome cancer-related fatigue
Step Six: Record Obtain all feedback from participants about the programme and their perceived experiences during and at the end of the implementation The Master of Counselling in Midwifery student besides the related subjects in her academic curriculum, she has attended in sexology counselling workshops so she has eligible certificate to handle the intervention

The participants also had the opportunity to express their experiences and feelings on a daily basis. This was done both individually and, in a group, online. As shown in Table 1, the fifth stage of the BETTER model is the educational stage. In this regard, the research team created appropriate educational packages in the form of video clips, images, and recorded voices. These were then delivered through WhatsApp. All content of the intervention program was created by the research team using the existing literature and then modified based on the steps of the model. The content validity of the intervention was then confirmed by ten academic staff from the faculty members' university.

Each session began with challenging questions and participants responded by brainstorming. Group members were encouraged to share their experiences with other group members.

The participants' answers were carefully checked by the group facilitator and the process of encouraging them to answer the questions continued until the codes obtained from the answers were saturated.

During the week, the participants kept their experiences in the form of daily notes as weekly assignments and shared them with the group members. The tasks consisted of recognizing the causes of fears, beliefs, disgust, shame and negative attitudes as threatening obstacles to sexual health, identifying the destroying factors of the couple's daily cooperation and intimacy, setting goals to achieve intimate relationships by practicing sharing feelings and emotions, trying to distinguish the barriers to accepting a positive body image, following self-regulated goals to value flexible sexual experiences. Participants had the opportunity to share their daily lived experiences, express barriers and facilitators to establishing the new learned behaviors, and search for solutions. Daily written feedback was provided by a trained researcher. If necessary, additional feedback and individual counseling could be provided through personal contacts. The women in the intervention group were asked to share the perceived information with their partners and to practice it with them.

All the feedback from the participants about the program and their life experiences during and at the end of the implementation were collected. In this regard, various suggestions regarding the positive points and benefits of the intervention program in their daily lives were collected through brainstorming in the form of feedback from the participants.

All the contents and issues of the intervention program were under the guidance of an expert psychiatrist who is a faculty member of the university and who acted as a supervisor in this master's thesis. The Masters student in midwifery counselling, in addition to the relevant subjects in her academic curriculum, has attended sexology counselling workshops, so she is qualified to carry out the intervention. The same interventionist led all groups.

### Statistical analyses

Data were first entered into SPSS Statistics for Windows, version 20 (SPSS Inc., Chicago, Ill., USA). The clinical and demographic characteristics of the two groups were compared using chi-squared test and independent samples t-test. Repeated measures analysis of variance (rANOVA) was used to assess the significance of the difference between the mean scores of the Female Sexual Function Index and Female Sexual Quality of Life of participants at three time points in each group. Before performing the rANOVA, the condition of equality of variances between groups was met by observing the assumptions and performing the necessary tests. In this regard, it should be mentioned that the amount of residual variance of the dependent variables was equal in all groups. Furthermore, the significance level (p-value) was considered to be less than 0.05. All analyses were performed by a researcher blinded to the data.

### Sample size calculations

The sample size was based on the study by Fatehi et al. [26] and using the mean difference formula for two independent groups of G power software, with the Sexual Quality of Life-Female (SQOL-F) questionnaire in the two intervention and control groups (mean1: 14.81, mean2: 21.42, SD1: 8.7, SD2: 6.7). Allowing for 20% attrition in each group, 45 participants were obtained (90 women in total)

$$n=\frac{{\left({Z}_{1-\frac{\alpha }{2}}+{Z}_{1-\beta }\right)}^{2}\times ({\sigma }_{1}^{2}+{\sigma }_{2}^{2})}{{({\mu }_{1}-{\mu }_{2})}^{2}}$$

## Results

Out of 156 breast cancer survivors assessed, 66 were excluded for not meeting the inclusion criteria: 25 lacked internet access, 13 had low literacy levels, 18 did not have their husband's permission, and 10 were unwilling to participate. Consequently, 90 eligible women were enrolled and completed the Sexual Quality of Life-Female (SQOL-F) and the Breast Cancer-Specific Sexual Function Index (FSFI-BC) questionnaires. They were then randomly assigned to either the control (*n* = 45) or intervention group (*n* = 45) using convenience sampling.

Subsequently, WhatsApp groups were established for each cohort. The intervention group participated in a 6-week BETTER model sexual counseling program, while the control group received standard self-care advice. Notably, there was full retention; all participants completed the 6-week program. Follow-up assessments were conducted post-intervention and at one month for both groups, as illustrated in Fig. 1.

Table 2 presents the socio-demographic and clinical characteristics of participants in both control and intervention groups. Statistical analyses, including the chi-squared test, Fisher's exact test, and independent samples t-test, showed no significant differences between the groups (*P* > 0.05). The mean duration since breast cancer diagnosis was comparable, with the intervention group at 3.16 years (SD = 1.64) and the control group at 3.22 years (SD = 1.78). A majority of the participants were homemakers, constituting 64.4% in the intervention group and 66.7% in the control group.

**Table 2:** Comparison of socio-demographic characteristics and some clinical issues of participants in control (*n* = 45) and intervention (*n* = 45) groups

Characteristics		Control	Intervention	Statistics
Quantitative variables		Mean (SD)	Mean (SD)	
Age		37.36 (6.51)	37.38 (7.49)	t = 0.82Df = 88 *P = 0.27
Menarche age		11.91 (1.20)	11.82 (0.94)	Z = 0.51 ^**#**^P = 0.6
Children(N)		1.18 (0.88)	1.51 (0.86)	t = 1.80 Df = 88 *p = 0.07
Duration of breast cancer diagnosed		3.22 (1.78)	3.16 (1.64)	t = 0.15, df = 98 *P = 0.87
Qualitative variables		N (%)	N (%)	
Place of residence	city	38 (84.4)	40 (88.9)	X^2^ = 0.38 Df = 1 ^**#**^*p* = 0.53
	village	7(15.6)	5 (11.1)	
Level of education	High school & Diploma	25 (55.6)	24 (53.3)	^**&**^P_fisher_ = 0.33
	Master's degree	17 (37.8)	19 (42.2)	
	Ph.D	3 (6.7)	2 (4.4)	
Women's job	Governmental Job	14 (31.1)	12 (26.7)	^**&**^P_fisher_ = 0.83
	Self-employment	2 (4.4)	3 (6.7)	
	Housekeeper	30 (66.7)	29 (64.4)	
Husband's education level	High school& Diploma	17 (37.8)	16 (35.5)	^**&**^P_fisher_ = 0.63
	Master'degree	26 (57.8)	24 (53.3)	
	Ph.D	2 (4.4)	5 (11.1)	
Husband’s job	Employee	11(24.4)	19 (42.2)	^**&**^P_fisher_ = 0. 31
	Manual worker	7 (15.6)	8 (17.8)	
	Self-employment	24 (53.3)	15 (33.3)	
	Retired	2 (4.4)	1 (2.2)	
	Unemployed	1 (2.2)	2 (4.4)	
Sufficient family income	Enough Relatively	4 (8.8)	3 (6.7)	^**&**^P_fisher_ = 0.83
	Enough	32 (71.1)	32 (71.1)	
	Insufficient	9 (20.0)	10 (22.2)	
Diseases stage	**1**	23 (51.1)	18 (40)	X^2^ = 1.12 Df = 1 ^**#**^*p* = 0.39
	**2**	22 (48.9)	27 (60)	

^*^Independent Samples T-Test, ^**#**^ Chi-Square, ^**&**^ Fisher Exact Test

The results of the rANOVA on the significance of the difference in the mean scores of the sexual quality of life and sexual function index in the two groups are presented in Tables 3 and Table 4. Three types of interaction effects are also examined in these tables. Based on the results, the interaction between time and intervention was found to be significant (*p* < 0.001). This indicates that there was a significant difference in the mean sexual quality of life and sexual function index scores between the two groups at different time points. In other words, the trend of the mean scores of the response variables was not similar in the two groups over time.

**Table 3:** The results of the repeated measures ANOVA comparing the mean scores of the Female Sexual Function Index (FSFI-BC) and its dimensions at three time points: before, immediately and one month after the intervention in the control and treatment groups

Variables	Effect	Source	SST	DF^a^	MS	F	*P*-value	Eta
Changes after cancer	Between	Group	1642.8	1	1642.8	157.54	< 0.001	0.64
	Within	Time	1172.1	2	586.0	108.0	< 0.001	0.55
		Group by Time	967.2	2	483.6	89.15	< 0.001	0.50
Desire/ Arousal	Between	Group	2454.1	1	2454.1	151.0	< 0.001	0.44
	Within	Time	1985.4	2	992.7	79.88	< 0.001	0.48
		Group by Time	1416.6	2	708.3	56.99	< 0.001	0.39
Lubrication	Between	Group	183.03	1	183.03	22.40	< 0.001	0.63
	Within	Time	107.77	2	53.88	12.55	< 0.001	0.49
		Group by Time	91.550	2	45.75	10.65	< 0.001	0.45
Orgasm	Between	Group	111.1	1	111.1	39.51	< 0.001	0.75
	Within	Time	48.93	2	24.46	11.42	< 0.001	0.47
		Group by Time	38.79	2	19.40	9.05	0.001	0.41
Pain	Between	Group	71.78	1	71.78	55.56	< 0.001	0.81
	Within	Time	59.44	2	29.42	14.49	< 0.001	0.53
		Group by Time	38.37	2	19.19	9.36	0.001	0.42
Satisfaction	Between	Group	1549.2	1	1549.2	119.5	< 0.001	0.58
	Within	Time	1281.1	2	640.6	80.14	< 0.001	0.50
		Group by Time	1086.3	2	543.2	73.04	< 0.001	0.45
Distress	Between	Group	2364.4	1	2364.4	159.5	< 0.001	0.65
	Within	Time	902.9	2	451.5	51.37	< 0.001	0.40
		Group by Time	1131.6	2	565.8	64.38	< 0.001	0.42
Global FSFI-BC	Between	Group	68,311.1	1	68,311.1	233.5	< 0.001	0.73
	Within	Time	43,440.2	2	21,720.1	122.3	< 0.001	0.58
		Group by Time	38,047.8	2	19,023.9	107.1	< 0.001	0.55

*SST* Sum of Squares, *DF* Degree of Freedom, *MS* Mean of Squares

^a^Mauchly's test is used to test whether or not the sphericity assumption is met in a repeated measures ANOVA. If the sphericity assumption is rejected, we adjust the degrees of freedom using the Greenhouse–Geisser correction for within-subjects’ effects

**Table 4:** The results of the repeated measures ANOVA comparing the mean scores of sexual quality of life-female (SQOL-F) at three time points: before, immediately and one month after the intervention in the control and treatment groups

Variables	Effect	Source	SST	DF^a^	MS	F	*P*-value	Eta
sexual quality of life-female	Between	Group	17,232.0	1	17,232.0	61.14	< 0.001	0.41
	Within	Time	13,107.4	1.42	9220.2	32.66	< 0.001	0.27
		Group by Time	12,070.4	1.42	8490.7	30.08	< 0.001	0.26

*SST* Sum of Squares, *DF* Degree of Freedom, *MS* Mean of Squares

^a^Mauchly's test is used to test whether or not the sphericity assumption is met in a repeated measures ANOVA. If the sphericity assumption is rejected, we adjust the degrees of freedom using the Greenhouse–Geisser correction for within-subjects’ effects

In this study, the main effect of time was also found to be significant, showing a statistically significant difference between the mean scores of sexual qualities of life and sexual function index at different time points in the intervention group (*p* < 0.001). The main effect of intervention also showed that there was a statistically significant difference in the mean sexual quality of life and sexual function index scores between the two groups, independent of the effect of time. In other words, it was concluded that the mean scores of sexual qualities of life and sexual function index were higher in the intervention group compared to the mean scores of the control group (*p* < 0.001). Considering the partial eta squared, the effect of the intervention had the highest interaction effect on both variables of female sexual quality of life (η2 = 0.0.41) and female sexual function index (η2 = 0.73).

**Fig. 2:**
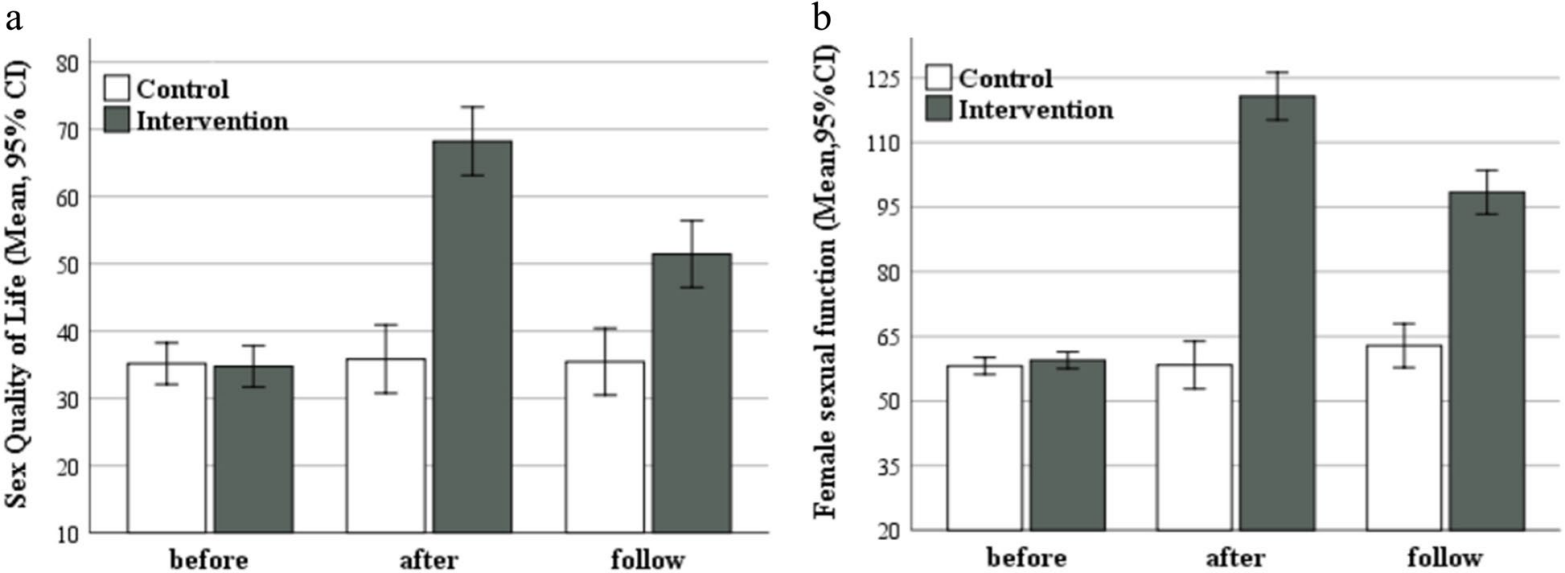
**a** Mean (95% confidence interval) of sex quality of life across 3 times and two groups. There was no significant mean difference between the two treatment and control groups before the intervention (*p*=0.85) whereas it had significantly increased in treatment group at immediately and 1 month after intervention (*p*<0.001). **b** Mean (95% confidence interval) of ***female sexual function*** across 3 times and two groups. There was no significant mean difference between the two treatment and control groups before the intervention (*p*=0.33) whereas it had significantly increased in treatment group at immediately and 1 month after intervention (*p*<0.001)

Figures 2a and b depict the temporal changes in the mean scores of the Sexual Quality of Life (SQOL) and Sexual Function Index (SFI) across both groups. The intervention group exhibited a significant improvement in both SQOL and SFI scores immediately after, and one month following, the counseling sessions compared to baseline. Notably, the peak mean scores for SQOL and SFI were recorded immediately post-intervention.

## Discussion

This randomized control trial study aimed to evaluate the effect of WhatsApp-based sexual counselling using the BETTER model on sexual quality of life and sexual function in breast cancer survivors. The results suggest that the intervention program had a significant effect on improving participants' sexual quality of life and sexual function. In the present study, the two intervention and control groups were homogeneous in terms of demographic, socioeconomic and some other factors listed in Table 2 that are known to influence sexual quality of life and sexual function [9, 15].

The results of the study by Hummel et al. showed that couples' sexual counseling based on cognitive behavioral therapy was effective in improving sexual performance, sexual intimacy, and satisfaction with sexual relationships in women with breast cancer [27], which was consistent with the results of the present study. Also, Jun et al. in South Korea reported the effect of a group counseling program on sexual satisfaction, marital intimacy, sexual interest, and sexual dysfunction in breast cancer patients after chemotherapy as an effective intervention to promote sexual health in this group of patients. They concluded that the program might be more effective if targeted to couples rather than survivors only, and if delivered earlier and over a longer period of time [28]. Almeida et al. emphasized sexual counselling sessions using the PLISSIT model for recognizing sexual issues, highlighting the sexual particularities of breast cancer survivors as a practical method [24]. In the other study, conducted by Fatehi et al., to investigate the effects of the Schover Sex Model as a multidimensional model on sexual activity led to improvements in sexual quality of life and sexual performance [26]. In line with the present study, a study conducted by Asgharipour et al. regarding the effectiveness of sexual counselling based on the BETTER model compared to the PLESSET model had a great impact on promoting the sexual outcomes of women undergoing mastectomy after breast cancer, and showed that the BETTER model is probably more suitable option for women with cancer [25]. A study by Olcer et al. also found that sexual counseling based on the BETTER model of sexual problems in women with breast cancer resulted in less pain during intercourse, improved lubrication, orgasm, and sexual performance. In addition, women who received counseling had a more positive mental image and a higher quality of sexual life. This study concluded that counseling based on the BETTER model is associated with increased sexual health awareness and decreased sexual distress in women with breast cancer [29]. Compared to the mentioned study, in the present study, since the counseling was online, the participants could not experience shame and disclosure about sexual issues, which are considered taboo in in-person counseling, and could express their concerns in the form of online counseling via WhatsApp and received the benefits of group discussion. Breast cancer is considered a stigma for breast cancer survivors [30], So, they try to hide their situation, and at the same time, they are looking for a trustworthy person to talk to about what they are feeling, to express their feelings and concerns, and even to talk about topics such as sexual issues, which are taboo in their culture. Cultural and religious factors can influence the psycho-sexual attitudes of women and their husbands [31, 32]. In Iranian culture, it is shameful and unacceptable for women to seek sexual relations by asking or showing interest. In addition, the husband's preferences and satisfaction with sexual relations are considered more important than the wife's satisfaction [33]. Couples also feel embarrassed to talk about sexual issues with their healthcare providers, especially in face-to-face sessions. This can leave sexual problems unrecognized and unresolved [34]. On the other hand, many studies have reported changes in the sex lives of breast cancer survivors, including a decrease in sexual relationships and a decrease in sexual desire [35–38]. Therefore, there is a need to create an open, truthful and accepting communication environment with women with breast cancer and their husbands within the healthcare system, and to help them meet their sexual health needs [39, 40].

The BETTER model as a counselling model based on principles and rules with a creative design, giving the participants the opportunity to express sexual problems and concerns, providing correct information about the anatomical and physiological aspects of sexual function, describing the individual's illness and its effects on sexual health, introducing reliable resources, challenging and creating a good mental preparation in the individual, clarifying the value of sexual relations with a focus on sexual intimacy and relying on psychological relaxation, improves performance and quality of life [41, 42].

In the present study, an open-ended, truthful, and accepting communication environment was attempted with breast cancer survivors through counseling via virtual networks. In this way, they found a very good opportunity to discuss their challenges in terms of body image concerns, intimacy and its barriers, concerns about sexual behavior, and struggles to find new sources of support. The intervention program attempted to enable breast cancer survivors to articulate their concerns and anxieties as barriers to better sexual quality of life and appropriate sexual performance, to disclose sexual problems, to identify erroneous beliefs and attitudes, to recognize practical and helpful solutions for rehabilitation and reclaiming sexual life. In addition, it sought to provide a space for participants to seek strategies for coping with the cancer journey and to feel a new sense of mastery and awareness in order to broaden perspectives on their own situation. The WhatsApp group counseling aimed to create a supportive atmosphere to provide a friendly environment while respecting the privacy of participants to express their unspoken words, fears, and doubts. On the other hand, it seems that the created group helped the participants to feel a deep sense of integration and belonging to an empathetic group, besides reducing feelings of loneliness, obtaining more empathy, being familiar with peers experiencing more difficult situation but hopefully looking for solutions to face sexual problems in the context of breast cancer. BETTER model in the intervention program tried to highlight the exercises in the program to help the participants to find new ways to improve the respectful communication, and consequently to transform their body image in a more positive side.

Finally, for clinical practice, it seems that the WhatsApp BETTER sex model intervention program could improve sexual function and sexual quality of life.

The WhatsApp-based intervention would provide greater access to the counsellor and participants anywhere and anytime. This mode of counselling would also be more cost-effective and participants would be able to openly express their worries and concerns without embarrassment, especially about topics such as sexual issues, which are considered taboo in some cultures.

Because the characteristics of the women allocated to the control and intervention groups were similar at baseline, the researchers could be more confident that any observed differences in outcomes between the groups were due to the intervention rather than confounding factors.

## Limitations

One of the main limitations of the study was that husbands were not included in the intervention group due to the taboo nature of sexual issues in the present study and the privacy of the participants. Group counselling for couples is suggested for future studies.

### Clinical implications

There is a huge amount of non-scientific information received by patients through virtual invalid resources, the reliability of which is doubtful. So it seems that health policy makers should be persuaded to produce educational packages based on reliable scientific texts regarding breast cancer survivors' health care and unresolved sexual complication and related stress, especially in the cultures where some of the life realities like sexual issues are considered to be taboo.

As the Whatsapp based BETTER sex model intervention had significant effect on sexual quality of life and sexual function. Therefore, it is recommended to be considered as one of the practical plans in the process of treatment and counselling of breast cancer survivors.

## Conclusion

The intervention program was seen as a successful model for improving female sexual quality of life and female sexual function in breast cancer survivors. In addition, during the COVID-19 pandemic and the ban on gatherings and laws based on reducing traffic, which led to limited access to medical and counselling services, the present study provided an opportunity to pay attention to the sexual issues. Thus, participants could access appropriate counselling to empower themselves to promote sexual quality of life and sexual function as their human rights.

## Data Availability

Data is provided within the manuscript or supplementary information files.

## Abbreviations

BETTER Sex Model: Bring up, Explain, Tell resources, Time, Educate, Record
SQOL-F: Sexual Quality Of Life-Female
FSFI-BC: Female Sexual Function Index- Breast Cancer
PLISSIT: Permission, Limited Information, Specific Suggestions, and Intensive Therapy
